# Physician’s Pocket Day-book, Journal, and Ledger

**Published:** 1889-12

**Authors:** 


### Physician's Pocket Day-book, Journal, and Ledger combined. By Dr. S. L. Kilmer, South Bend, Indiana. Price, $2.00.

As its name implies, this book is a neat, concise, and complete volume, bound in Russia, seven and one-fourth inches long, four inches wide, and five-eighths inches thick, being thus no longer than a common pocket-book. It is properly ruled into spaces which are duly labeled, and is so perfect in its arrangement that running accounts can be kept with four hundred individuals, together with unsettled accounts of preceding years, so that the physician may at all times be prepared to settle with debtors because he has their accounts always with him. This alone will save to the physician many times the cost of the book. Its use also saves much labor and time in keeping accounts, as the one entry completes the whole work of Day-book, Journal, and Ledger. It is well gotten up, and printed on excellent paper, and is an excellent assistant to every physician.

